# The comparison of the effects of nano-silymarin and silymarin on high-fat diet-induced fatty liver of adult male rats

**DOI:** 10.22038/AJP.2024.23734

**Published:** 2024

**Authors:** Reza Mohebbati, Mohammad Amin Momeni-Moghaddam, Rokhsareh Asghari, Abbasali Abbasnezhad, Alireza Ebrahim Zadeh Bideskan, Davoud Salarbashi, Nasim Khajavian

**Affiliations:** 1 *Department of Physiology, Faculty of Medicine, Gonabad University of Medical Sciences, Gonabad, Iran*; 2 *Clinical Biochemistry, Faculty of Medicine, Social Determinants of Health Research Center, Gonabad University of Medical Sciences, Gonabad, Iran*; 3 *Department of Anatomy and Cell Biology, School of Medicine, Mashhad University of Medical Sciences, Mashhad, Iran*; 4 *Department of Nutrition, Clinical Biochemistry and Food Sciences, Faculty of Medicine, Gonabad University of Medical Sciences, Gonabad, Iran*; 5 *Department of Epidemiology and Biostatistics, School of Health, Social Determinants of Health Research Center, Gonabad University of Medical Sciences, Gonabad, Iran*

**Keywords:** Silymarin, Nano-silymarin, Non-alcoholic fatty liver

## Abstract

**Objective:**

Considering the high prevalence of non-alcoholic fatty liver disease and its complications, this study aims to determine and compare the effect of nano-silymarin and silymarin on non-alcoholic fatty liver in rats.

**Materials and Methods:**

Rats were divided into 5 groups: Control, high-fat diet, high-fat diet and atorvastatin, high-fat diet and silymarin, and high-fat diet and nano-silymarin. After 12 weeks, blood samples were taken to measure cholesterol, triglyceride, HDL, LDL, ALT and AST. The animals were killed and the liver tissue was removed to examine the histopathological changes.

**Results:**

Feeding with a high-fat diet caused a significant increase in cholesterol, triglyceride and LDL-C in serum of rats compared to the control. Nano-silymarin and silymarin could significantly reduce serum triglyceride compared to negative group but the reduction of cholesterol, LDL-C, AST and ALT by nano-silymarin was not significant as compared to silymarin. The liver histology evaluation mainly showed that in the group receiving nano-silymarin, a significant decrease in the percentage of fat vacuoles and degree of steatosis was observed compared to the negative group. In the positive group, the percentage of fat vacuoles and the degree of steatosis showed a significant decrease compared to the negative group. Group receiving atorvastatin showed a greater protective effect than silymarin and nano-silymarin.

**Conclusion:**

The use of nano-silymarin similar to silymarin in rats treated with a high-fat diet led to a decrease in the percentage of fat vacuoles and the degree of hepatic steatosis mainly and can be used to prevent non-alcoholic fatty liver disease.

## Introduction

Non-alcoholic fatty liver disease (NAFLD) is known as one of the most common types of chronic liver diseases worldwide and is a reversible disease caused by the accumulation of large amounts of fat (triglyceride) in liver cells. In fact, NAFLD is a chronic liver disease that includes a wide range of clinical symptoms (from asymptomatic fatty liver to severe inflammation of the liver with fibrosis and sometimes cirrhosis). In these patients, insulin resistance and cardiovascular diseases are also highly prevalent (Targher et al., 2007). NAFLD was first recognized and introduced in 1980. Obesity, increased blood sugar, type 2 diabetes and increased blood lipids are among the most important causes of NAFLD (Norouzi et al., 2022; Sáez-Lara et al., 2016). Medicinal plants have critical role in the many disorders mainly by anti-inflammatory (Heravi et al., 2018) and anti-oxidant (Khazdair et al., 2016; Parhizgar et al., 2016) mechanisms.

Silymarin (S) derived from the *Silybum marianum* has a protective effect on liver cells, and previous studies on this plant have shown its effect on lowering plasma cholesterol and liver cholesterol content. It also has therapeutic effects on liver cirrhosis, diabetes, blood lipids, cataracts, osteoporosis and –cancer (Ghosh et al., 2011; Lirussi et al., 2002). One of the important challenges in the development of Silymarin formulation is its poor aqueous solubility (Jacobs et al., 2002). The Silymarin has many biological properties, but its bioavailability and therapeutic efficiency are limited by this issue (Ahmad et al., 2015; Hsu et al., 2012). About 50% of Silymarin is absorbed through the gastrointestinal tract (Javed et al., 2011).

Micelle nanoparticles were used in this study. Micelles are nanometer-sized particles that have a phospholipid monolayer and a hydrophobic lipid core. Many amphiphilic molecules have a polar hydrophilic group and a nonpolar hydrophobic group (Torchilin, 2007). The important feature of micellar drugs is the increase in their ability to pass from the cell membrane. In addition, micelles have other advantages such as increased saturation solubility and dissolution speed and excellent adhesion to biological surfaces (Junghanns et al., 2008; Kakran et al., 2015). Therefore, in this study, the comparative effects of nano-silymarin and Silymarin in NAFLD in rats were investigated.

## Materials and Methods

The present study was conducted on 35 adults male Wistar rats purchased from the laboratory animal center of Gonabad University of Medical Sciences weighing 270 to 330 grams. First, the animals were kept for two weeks in order to adapt to the environmental conditions with an average temperature of 23±2°C and observing the light/dark cycle for 12 hr each with free access to food and water. All procedures were performed based on the animal ethics. The ethical code is IR.GMU.REC.1400.116.

Rats were randomly divided into 5 groups (n=7): Group 1 (standard deit group): fed with standard food + 1 ml of normal saline daily by gavage for 12 weeks. Group 2 (high-fat diet group): fed with a high-fat diet without treatment + 1 ml of normal saline daily by gavage for 12 weeks. Group 3 (atorvastatin group): fed with high-fat diet + 20 mg/kg of atorvastatin daily by gavage for 12 weeks (Farrag et al., 2018). Group 4 (silymarin group): fed with high-fat diet + 100 mg/kg Silymarin daily by gavage for 12 weeks (Hajizadeh Moghaddam et al., 2017). Group 5 (nano-silymarin group): fed with high-fat diet + 100 mg/kg of NS daily by gavage for 12 weeks (Hajizadeh Moghaddam et al., 2017).

To prepare a high-fat diet, 45% of standard rodent food, 49.8% fat (melted sheep tail fat), 0.2% cholesterol powder and 5% wheat flour (to improve the taste of the diet) were mixed. Calories and energy required to induce fatty liver are appropriate (Ghaeni Pasavei et al., 2021).

**Preparation of nano-silymarin**

**Figure 1:**
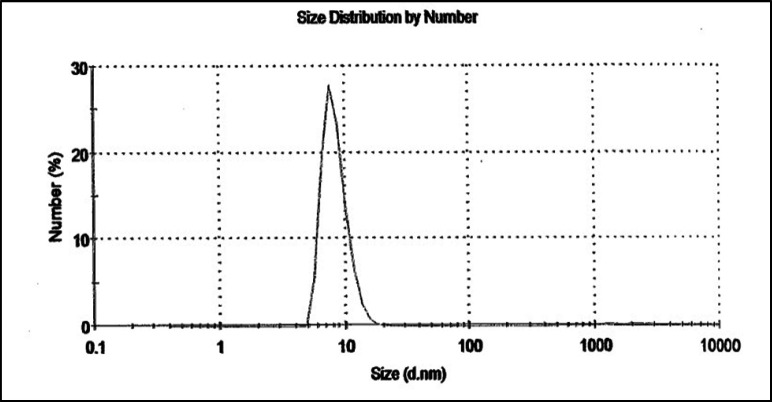
The DLS report for nano-silymarin sample

The colloidal solution of NS in amber glass was prepared from Exirnano Sina Company (the Dynamic Light Scattering (DLS) report is shown in Figure 1). DLS measures the hydrodynamic diameter of nanoparticles in solution and provides information on the aggregation state of nanoparticles in solution. The NS solution was kept away from light at room temperature. Considering that the concentration of the solution was 10%, the amount required for daily use was diluted with 5% dextrose serum in a ratio of 1: 100 mg/kg of this solution was used daily by gavage for each animal.

After 12 weeks, after 12 hr of fasting, rats were weighed again and then put under anesthesia with 100 mg/kg of ketamine and 10 mg/kg of xylazine intraperitoneally, and after cutting in the abdomen, intracardiac blood samples were taken. After that, the liver tissue was removed and after washing with normal saline, a part of it was placed in a container containing 10% formalin to check histopathological changes.

**Measurement of biochemical factors**

Blood samples obtained for serum preparation were centrifuged (4000 rpm, 4°C for 15 min) and serum samples were kept in a freezer at -20°C until analysis. Measurement of liver enzymes (Aspartate transaminase (AST) and alanine aminotransferase (ALT)) and lipid profile (cholesterol, triglyceride (TG) and HDL-C) of the serum was done photometrically using standard kits (Pars Azmoon, IR). The Friedwald equation was also used to determine the amount of LDL-C in the serum.

**Liver histopathology study**

The tissue samples were molded in paraffin blocks. Then, using a microtome, 5 micrometer thick slices were prepared from the tissues and stained with hematoxylin and eosin method. Stained tissue sections in different groups were photographed using an optical microscope equipped with a camera (Olympus BX51-TF, Japan) at x40 magnification. The histopathological changes of the liver in the form of hepatocyte fat changes were scored based on severity of the lesions. Tissue changes based on the amount of fat accumulation in the liver tissue of different groups were evaluated as the percentage of hepatocyte cells with symptoms of the presence of fat (Efati et al., 2016).

**Statistical analysis**

Data is presented as Mean±SD. Normality was confirmed by the Kolmogorov-Smirnov test, one-way analysis of variance statistical test was used to compare groups, and Tukey's post hoc test was used. Also, the pathological stages and the grading results of the groups in terms of fat accumulation in hepatocytes were compared using the Kruskal-Wallis statistical test and the Mann-Whitney post hoc test. For this evaluation SPSS v.16 was used. Level of significance was considered less than 0.05.

## Results

The results showed that the weight of rats in the negative group increased significantly compared to the control group (p<0.05). The weight of rats in the positive group significantly decreased compared to the negative group (p<0.05). The weight of rats in the Silymarin receiving group decreased compared to the negative group, but it was not significant. Also, the weight of rats in the group receiving nano-silymarin decreased compared to the negative group, but it was not significant (Table 1).

**Table 1:** Comparison of the weight of rats before and after the intervention in the studied groups.

Groups Weight	Standard diet	High-fat diet	atorvastatin	Silymarin	Nano-silymarin
Before	300±23	304±21	307±19	306±12	301±17
After	381±37	471±14**	390±67#	416±46	406±50

Data are presented as Mean±SD. **p<0.01 compared to the control group, #p<0.05 compared to the negative group.

**Figure 2:**
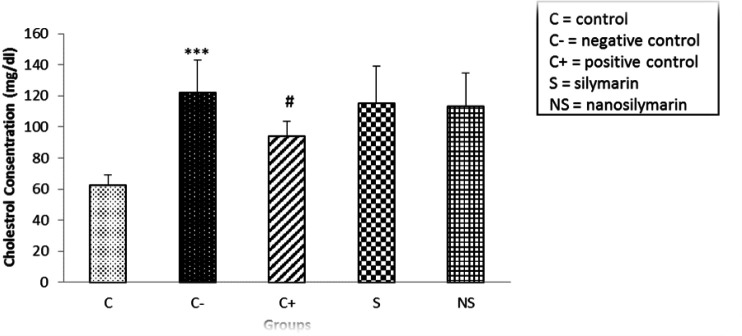
Comparison of mean serum cholesterol levels in the studied groups. Data is presented as Mean±SD. ***p<0.001 compared to the control group, #p<0.05 compared to the negative group.

Comparing the serum cholesterol level of rats between the control and negative groups showed a significant increase of this factor in the negative group (p<0.001). Comparison of the amount of cholesterol between the positive group and the negative group showed a significant decrease in the positive group (p<0.05). Comparison of serum cholesterol levels in the groups receiving silymarin (p<0.001) and nano-silymarin showed a significant increase compared to the control group (p<0.001). Also, the amount of cholesterol in the groups receiving Silymarin and nano-silymarin was reduced compared to the negative group, which was not significant (p>0.05). About this parameter, no significant difference was observed between the group receiving S and the group receiving nano-silymarin (p>0.05) (Figure 2).

**Figure 3:**
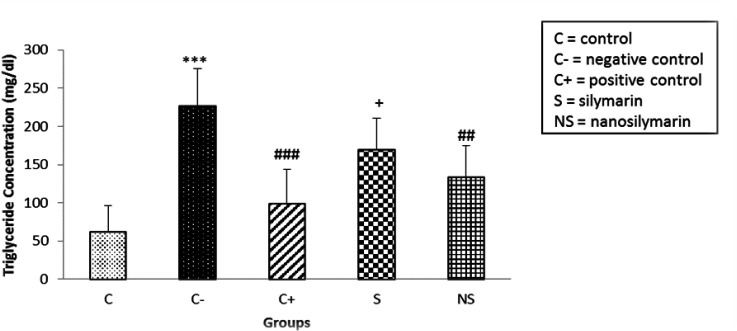
Comparison of mean serum triglyceride levels in the studied groups. Data is presented as Mean±SD. ***p<0.001 compared to the control group, ##p<0.05 and ###p<0.001 compared to the negative group, +p<0.05 compared to the positive group.

The results showed that the TG level in the negative group increased significantly compared to the control (p<0.001). Comparing the TG level between the positive and negative groups showed a significant decrease in the positive group (p<0.001). Also, the comparison of the mean TG between the negative group and nano-silymarin receiving group shows a significant decrease in the nano-silymarin receiving group (p<0.01). Receiving Silymarin led to a decrease in serum TG level, which was not significant compared to the negative group (p>0.05). In comparing the mean TG between the positive group and those receiving S, the 

positive group showed a significant decrease in TG compared to Silymarin (p<0.05) (Figure 3).

**Figure 4:**
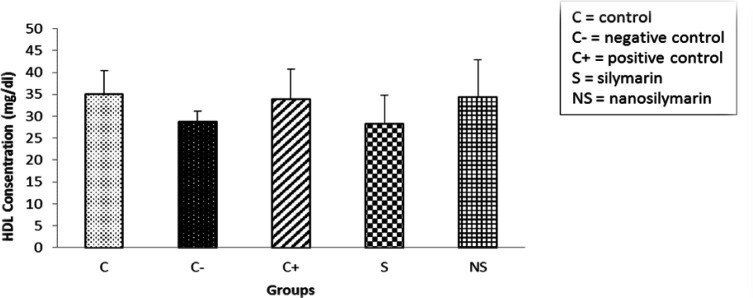
Comparison of mean serum HDL-C levels in the studied groups. Data is presented as Mean±SD. There was no significant difference among the groups.

The amount of HDL-C in the negative group decreased compared to the control group, but this decrease was not significant (p>0.05). HDL-C levels increased in the group receiving NS compared to the negative group, but this increase was not significant (p>0.05) (Figure 4).

**Figure 5:**
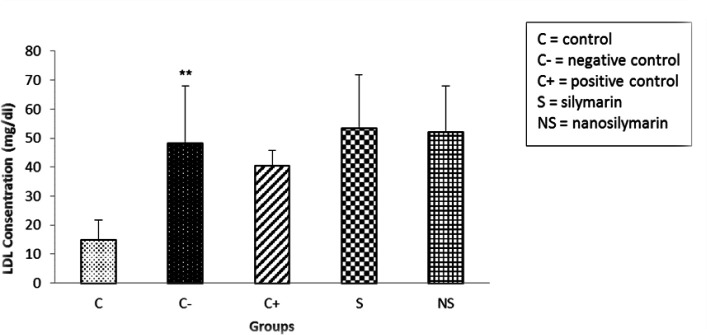
Comparison of mean serum LDL-C levels in the studied groups. Data is presented as Mean±SD. **p<0.01 compared to the control group.

The results showed that serum LDL-C levels between the control and negative groups significantly increased in the negative group (p<0.01). Comparison between the positive and negative groups showed a decrease in LDL-C in the positive group, which was not significant (p>0.05). However, receiving Silymarin and nano-silymarin did not lead to a decrease in LDL-C serum level compared to the negative group (Figure 5).

**Figure 6:**
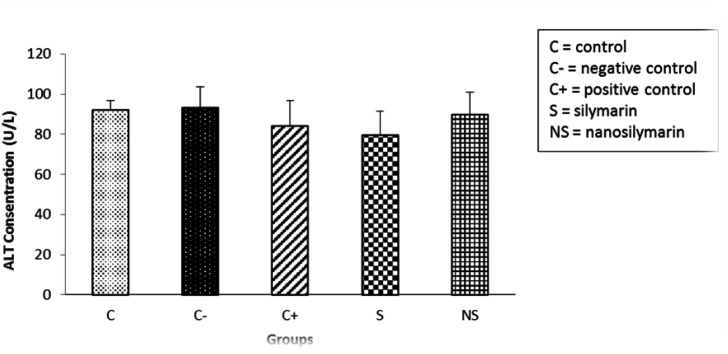
Comparison of mean serum ALT levels in the studied groups. Data is presented as Mean±SD. There was no significant difference among the groups.

The results showed that there were no significant changes in the average serum activity of ALT enzyme between the studied groups (Figure 6).

**Figure 7:**
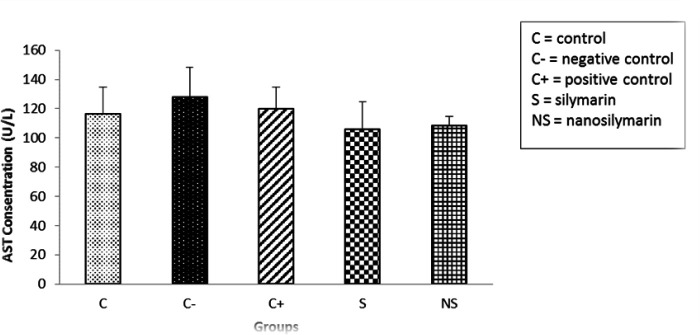
Comparison of mean serum AST levels in the studied groups. Data is presented as Mean±SD. There was no significant difference among the groups.

The results showed that the AST values in the negative group increased compared to the control group but there was no significant difference (p>0.05). The amount of liver enzymatic activity in the groups receiving atorvastatin, S and NS decreased compared to the negative group, but this decrease was not significant (Figure 7).

**Figure 8:**
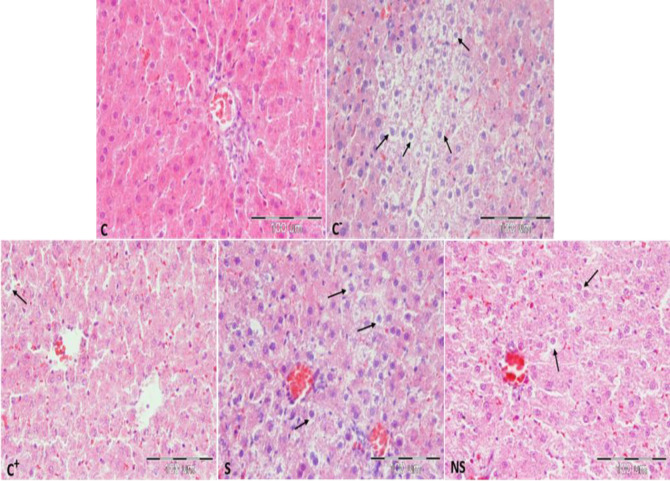
Photomicrograph of liver tissue stained with hematoxylin-eosin staining. (C) Control group: hepatocytes and liver tissue structure are normal. (Negative group) Group fed with a high fat diet: Fat change is characterized by the formation of vesicles. (Positive group) high fat diet group + atorvastatin: the liver tissue is normal and a significant decrease in fat vacuoles is seen compared to the high fat group. (S) High fat diet + silymarin group: fat vesicles are observed in the liver tissue, the change in liver fat is not statistically significant compared to the high-fat diet group. (NS) high fat diet + nano-silymarin group: the amount of fat vesicles is mild and can be seen scattered, and the liver tissue shows a normal appearance. Arrows indicate hepatocytes containing fat vacuoles. x40 magnification.

Microscopic results showed that the liver tissue had natural structure in the control rats. However, the high -fat diet caused fat accumulation in hepatocytes as small and large vacuoles. The Silymarin intake did not significantly reduce the percentage of fat vacuoles or the degree of steatosis compared to negative group. Whereas in nano-silymarin recipients a significant decrease in the percentage of fat vacuoles and the degree of steatosis was observed. In the positive group, the percentage of fat vacuoles and steatosis also showed a significant decrease compared to the negative group. Also, there was no significant difference between positive groups and NS in terms of changes in fat vacuoles and steatosis, although atorvastatin receiving group showed a greater protective effect than Silymarin and nano-silymarin (Figure 8).

Microscopic examinations showed that the liver tissue in the control group rats had a normal structure. This is while the high-fat diet caused the accumulation of fat in the hepatocytes in the form of small and large vacuoles between control and negative groups (p<0.01). Receiving S did not cause a significant decrease in the percentage of fat vacuoles and the degree of steatosis compared to negative group (p>0.05).

While in the group receiving nano-silymarin, a significant decrease in the percentage of fat vacuoles and degree of steatosis was observed compared to the negative group (p<0.05). In the positive group, the percentage of fat vacuoles and the degree of steatosis showed a significant decrease compared to the negative group (p<0.01). Also, in the comparison of fat between the positive group and NS groups, no significant difference was observed in terms of changes in the percentage of fat vacuoles and the degree of steatosis, although the group receiving atorvastatin showed a greater protective effect than S and NS (Tables 2 and 3).

**Table 2:** Comparison of the median and interquartile range of NAFLD grading of rats in the negative control group compared to the control group (C).

Groups		negative group		C
Median		4		1
interquartile range		1		0
p-value		0.01		

**Table 3:** Comparison of median and interquartile range of NAFLD grading of rats in treatment groups compared to negative control group.

Groups	negative group	positive group	S	NS
Median	4	2	3	2
interquartile range	1	1.25	1	1.25
p-value		0.01	0.33	0.05

## Discussion

In a study conducted by "Khoushbatin et al." on 102 patients and 102 healthy people in East Azerbaijan, obesity in patients with fatty liver was significantly more common (Khoushbaten et al., 2009). In another study after 4 weeks, mice that received a high-fat diet had higher weight (Esposito et al., 2009). The American National Institute of Health has defined hyperlipidemia as an increase in the serum level of TG, total cholesterol, and LDL-C and a decrease in the level of HDL-C (Athyros et al., 2011). These fat disorders may be primary (hereditary) or secondary (Kusters et al., 2013). Secondary hyperlipidemia is caused by unhealthy diets, alcohol consumption, estrogen therapy, or diseases such as diabetes mellitus, hypothyroidism, and chronic kidney disease (Mahamuni et al., 2012).

Several studies have shown that animals fed with a high-fat diet are at higher risk of metabolic disorders such as hyperlipidemia, hepatic steatosis, and oxidative stress. A high-fat diet induces hyperlipidemia in mice, which is characterized by decreased levels of antioxidant enzymes, increased cholesterol levels, and damage to liver tissue (Li et al., 2010; Pérez-Echarri et al., 2009; Venkateshan et al., 2016). 

In this study, consumption of high-fat diet could increase the amount of liver enzymes (AST and ALT) compared to the control group, but this increase was not significant. In a study conducted by Molleston et al in 2014 it was found that 19% of children with confirmed NAFLD had normal ALT levels (Molleston et al., 2014).

In a study conducted by Saeidian et al. in 2019, it was shown that in alcoholic fatty liver, the increase of AST enzyme is more than ALT enzyme but in NAFLD, the increase of ALT enzyme is more than AST (Saeidian et al., 2019). In another study conducted by Khoushbatin et al. on 102 patients and 102 healthy individuals in East Azerbaijan, the results showed that the increase of AST and ALT significantly was higher in the patient group than in the control group (Khoushbaten et al., 2009).

As mentioned, the lack of significant changes in liver enzymes in our groups was in contrast with the researches of Khoushbatin et al. and Saeidian et al. Among the possible causes, we can refer to the different methods of creating NAFLD in our study compared to other studies conducted in this field. In the study conducted by Dehbashi, the high-fat diet was prepared in such a way that 15% of animal fat, 4% of cholesterol and 1% of cholic acid were added to the basic food of rodents, which was different from the composition used in our study. On the other hand, AST enzyme is naturally found in different types of tissues such as the liver, heart, muscle, kidney and brain, and this makes us consider ALT more specific than AST for the liver, so factors such as differences in the sample diet and also the effects of inducing fatty liver along with gavage of drugs may be effective on other organs that release this enzyme (Dehbashi et al., 2021).

In the current study, microscopic examinations showed that the liver tissue in the control group rats had a normal structure, while the high-fat diet caused the accumulation of fat in the hepatocytes in the form of small and large vacuoles. In a study conducted by Ghaeni et al. in 2021, the pathology results showed that the diet high fat leads to grade 1 fatty liver (Ghaeni Pasavei et al., 2021). The results of this study also confirm the effect of high-fat diet in causing NAFLD.

In a study conducted by Iranikhah et al. the result showed that S consumption could not statistically significantly change the mean of HDL-C, LDL-C and TG and the weight of patients compared to the control group, which was in accordance with the findings of our research (Iranikhah et al., 2017).

In another study conducted by Safian et al. the result showed that the lipid profile parameters in the group treated with nano-micelle were significantly reduced compared to the group treated with Silymarin (Safian Isfahani et al., 2021). In our study, nano-silymarin was able to reduce TG and cholesterol more than Silymarin. The nano-silymarin and silymarin only reduced triglyceride level and there was not significant difference between the two drugs probably due to some biochemical structure properties. 

In the current study, liver enzyme AST and ALT in the negative group did not increase significantly compared to the control group. Taking Silymarin and nano-silymarin could reduce AST and ALT, which was not significant. Also, no significant difference was found between the results of liver enzymes in rats receiving Silymarin and nano-silymarin.

Also, microscopic studies showed that receiving silymarin and nano-silymarin cause a significant decrease in the percentage of fat vacuoles and the degree of steatosis compared to the high-fat diet group. However, results showed that there is not significant difference between these groups. Various reasons can cause this conclusion, including insufficient drug dose or environmental conditions and animal breed

It is important to mention that in all the studies in which the studied population was with nonalcoholic steatohepatitis characteristics and their AST and ALT levels were higher than normal, a decrease in liver enzymes was observed due to the consumption of silymarin, while in our study, rats received Silymarin and nano-silymarin with the high-fat diet.

Based on the results, The use of nano-silymarin similar to silymarin in rats treated with a high-fat diet led to a decrease in the percentage of fat vacuoles and the degree of hepatic steatosis mainly and can be used to prevent non-alcoholic fatty liver disease. In addition, due to the lack of significant difference in the amount of fat accumulation in hepatocytes in rat treated with nano-silymarin and atorvastatin, the use of nano-silymarin can be used to prevent and treat fatty liver.
